# The Outcomes of Laparoscopic Sleeve Gastrectomy for the Treatment of Obesity: A Single-Center Experience

**DOI:** 10.7759/cureus.48662

**Published:** 2023-11-11

**Authors:** Layan Khushaim, Abdulrahman G Alhazmi, Ibrahim S Omayer, Majdah F Alqahtani, Alhareth S Alsalama, Afnan W Alsulami, Firas A Hasaballah, Abdullah M Alzahrani, Shaker A Majrashi

**Affiliations:** 1 General Surgery, King Fahad General Hospital, Jeddah, SAU; 2 Medicine, Ibn Sina National College, Jeddah, SAU

**Keywords:** bariatric surgery, obesity, saudi arabia, weight reduction, outcomes, gastric sleeve surgery, sleeve

## Abstract

Background: Obesity is a long-standing health issue in Saudi Arabia, known to be associated with various complications. The management of obesity encompasses both non-surgical and surgical interventions, such as sleeve gastrectomy. Although sleeve gastrectomy is one of the effective options for individuals with morbid obesity, it is not without potential complications. This study aims to examine the outcomes of patients who underwent laparoscopic sleeve gastrectomy.

Methods: A descriptive, retrospective study was conducted on adult patients who underwent laparoscopic sleeve gastrectomy at King Fahad General Hospital in Jeddah, Saudi Arabia, between January 2017 and July 2022.

Results: Among the 561 adult patients in the study, 53.5% were classified as having class III obesity, and 74.2% had comorbidities. Complications observed following the procedure included leaking (3.2%), symptomatic gallstone disease (2.9%), internal hernia (1.8%), and readmission (2.1%). There were no cases of bleeding, aspiration pneumonia, or mortality reported. Leakage and gallstone disease were more prevalent among patients classified as class I and II obesity, respectively, while internal hernia and readmission were more frequently observed in patients with class III obesity.

Conclusion: Laparoscopic sleeve gastrectomy is a viable procedure for managing obesity, as it is associated with minimal complications and no recorded mortality.

## Introduction

Obesity represents a significant global health concern in the present era. In 2016, it was estimated that the worldwide population of obese individuals exceeded 650 million, accounting for approximately 13% of the global population [1]. In Saudi Arabia, obesity has emerged as a prominent health issue [2]. The accumulation of excess body weight gives rise to various complications, resulting in the progressive deterioration of bodily organs and systems. Notably, abdominal obesity stands out as a significant risk factor for metabolic syndrome, a condition encompassing dyslipidemia, hypertension, and insulin resistance. Furthermore, individuals with metabolic syndrome are predisposed to the development of stroke, type 2 diabetes, and myocardial infarction, which contributes to increased mortality rates [1].

Addressing obesity entails restoring the balance between caloric intake and expenditure while addressing the associated comorbidities [3]. Conservative approaches to weight reduction include increased physical activity, pharmacotherapy, and dietary modifications. However, these interventions may prove inadequate in achieving optimal weight loss for certain patients [3]. Consequently, there has been a growing number of obese patients seeking surgical treatment as a result of the failure of non-surgical measures to yield desired outcomes in terms of weight reduction, improvement of comorbidities, functional capacity, and quality of life [4].

Presently, the most common bariatric surgical procedures include laparoscopic sleeve gastrectomy (LSG), adjustable gastric band placement, and Roux-en-Y gastric bypass [5]. LSG is an effective option for treating morbid obesity, as it leads to substantial and sustained weight loss. Additionally, it often results in partial or complete remission of obesity-related comorbidities [6-9]. Despite the effectiveness of LSG, it is associated with certain complications, as with other bariatric surgeries. However, LSG exhibits a lower incidence of postoperative complications than Roux-en-Y gastric bypass [3]. This study evaluated the outcomes of patients undergoing LSG, specifically on mortality rates and postoperative complications.

## Materials and methods

Study design, settings, and subjects

The present study employed an observational, retrospective methodology and was carried out at King Fahad General Hospital in Jeddah, Saudi Arabia, from January 2017 to July 2022. The study included adult individuals who underwent LSG while excluding participants below 18 years of age, those who underwent laparotomy, and individuals who lost follow-up during the initial year after the procedure. A comprehensive data sheet was created to gather detailed patient information, encompassing demographic characteristics, medical history, and treatment outcomes.

Statistical analysis

Data were simultaneously entered into a preform and updated. It was entered into Microsoft Excel (MS Office 2010). The data were analyzed using Statistical Product and Service Solutions (SPSS, version 22.0) (IBM SPSS Statistics for Windows, Armonk, NY). Descriptive analysis included the computation of frequencies and percentages. The categorical variables were represented as numbers, whereas quantitative variables were described using means ± SD. Kruskal-Wallis tests were used to distinguish between independent variables, and chi-squared tests were used to differentiate between variables. P-value < 0.05 should be considered for significance.

## Results

This investigation comprised a cohort of 561 patients who underwent LSG. The demographic characteristics of these patients can be found in Table 1. The average age of the patients was 39.1 ± 12.9 years. Notably, a substantial disparity existed in the gender distribution, with a female patient population of 378 (67.4%) individuals. In terms of body mass index (BMI), the average ± standard deviation (SD) among the patients was 41.1 ± 7. Furthermore, a significant discrepancy was observed across the BMI classifications, as individuals with class III obesity accounted for the most significant proportion of patients, numbering 300 (53.5%). Moreover, the vast majority of patients were non-smokers, comprising 523 individuals (93.2%).

**Table 1:** Demographic data of patients who underwent laparoscopic sleeve gastrectomy. Data are represented as mean ± SD (median) or number and percentage, body mass index (BMI), diabetes mellitus (DM), hypertension (HTN), heart failure (HF), obstructive sleep apnea (OSA), gastroesophageal reflux disease (GERD), chi-square.

Data (n=561)		Result
Age, years (mean)		39.1±12.9 (38.0)
Gender	Female	378 (67.4%)
	Male	183 (32.6%)
BMI average (standard deviation)		41.1±7.0 (40.0)
Classes of Obesity	Overweight (25-29.9)	(0.5%)
	Class I obesity (30-34.9)	92 (16.4%)
	Class II obesity (35-39.9)	166 (29.6%)
	Class III obesity ≥ 40	300 (53.5%)
Smoking	Yes	38 (6.8%)
	No	523 (93.2%)

Table 2 presents the comprehensive medical histories of the individuals included in the study. The primary medical conditions observed were hypertension, affecting 228 (40.6%) individuals; obstructive sleep apnea (OSA), which was identified in 178 individuals (31.7%); dyslipidemia, observed in 177 (31.6%) individuals; and diabetes mellitus, present in 147 (26.2%) individuals. Conversely, the least prevalent medical conditions were chronic obstructive pulmonary disease (COPD), affecting only three (0.5%) individuals; asthma, identified in 21 (3.7%) individuals; heart failure, observed in 14 (2.5%) individuals; atrial fibrillation (A-fib), present in four (0.7%) individuals; stroke, affecting five (0.9%) individuals; chronic kidney disease (CKD), observed in eight (1.4%) individuals; hemodialysis, required by one (0.2%) individual; and gastroesophageal reflux disease (GERD), identified in 15 (2.7%) individuals based on symptoms; however, their gastroscopy studies were normal.

**Table 2:** Past medical history of patients who underwent laparoscopic sleeve gastrectomy. Data are represented as number and percentage, chronic obstructive pulmonary disease (COPD), atrial fibrillation (A-Fib), coronary artery disease (CAD), chronic kidney disease (CKD), and peripheral vascular disease (PVD), chi-square.

Medical disorder		Number (%)	
DM	Yes	147 (26.2%)	
	No	414 (73.8%)	
HTN	Yes	228 (40.6%)	
	No	332 (59.2%)	
Dyslipidemia	Yes	177 (31.6%)	
	No	384 (68.4%)	
COPD	Yes	3 (0.5%)	
	No	558 (99.5%)	
Asthma	Yes	21 (3.7%)	
	No	540 (96.3%)	
HF	Yes	14 (2.5%)	
	No	547 (97.5%)	
A-fib	Yes	4 (0.7%)	
	No	557 (99.3%)	
OSA	Yes	178 (31.7%)	
	No	383 (68.3%)	
Stroke	Yes	5 (0.9%)	
	No	556 (99.1%)	
CAD	Yes	3 (0.5%)	
	No	558 (99.5%)	
CKD	Yes	8 (1.4%)	
	No	553 (98.6%)	
Hemodialysis	Yes	1 (0.2%)	
	No	560 (99.8%)	
GERD	Yes	15 (2.7%)	
	No	546 (97.3%)	

Table 3 documents 12 patient outcomes, and the notable absence of reported incidents included bleeding, aspiration pneumonia, and mortality. The prevalence of the remaining nine outcomes varied significantly, with each representing a relatively small proportion. Notably, the most frequently reported outcome was leaking, occurring in 18 (3.2%) cases, followed by gallstone disease affecting 16 (2.9%) patients. Additionally, readmission was required for 12 (2.1%) individuals, while stenosis was observed in only two (0.4%) cases. Venous thromboembolism (VTE) affected three (0.5%) cases, marginal ulceration was reported in 1 (0.2%) case, and perforation occurred in one (0.2%) case.

**Table 3:** Outcomes of laparoscopic sleeve gastrectomy surgery. Data are represented as numbers and percentages and chi-square.

Outcomes		Number (%)
Leaking	Yes	18 (3.2%)
	No	543 (96.8%)
Stenosis, twists, or kinks	Yes	2 (0.4%)
	No	559 (99.6%)
Bleeding	Yes	0 (0%)
	No	561 (100%)
Venous thromboembolism	Yes	3 (0.5%)
	No	558 (99.5%)
Gallstone disease	Yes	16 (2.9%)
	No	545 (97.2%)
Marginal ulceration	Yes	1 (0.2%)
	No	560 (99.8%)
Perforation	Yes	1 (0.2%)
	No	560 (99.8%)
Small bowel obstruction	Yes	2 (0.4%)
	No	559 (99.6%)
Internal hernia	Yes	10 (1.8%)
	No	551 (98.2%)
Aspiration pneumonia	Yes	0 (0%)
	No	561 (100%)
Readmission	Yes	12 (2.1%)
	No	549 (97.9%)
Mortality	Yes	0 (0%)
	No	561 (100%)

The examination of patient outcomes utilizing BMI revealed no significant differences among the three classifications of obesity (as indicated in Table 4). Nevertheless, it was observed that leaking occurred more frequently in patients with class I obesity in comparison to the other two classes. Instances of gallstone disease and perforation were more commonly reported in patients classified as class II, while VTE, marginal ulceration, internal hernia, and readmission were more prevalent among individuals classified as class III obesity.

**Table 4:** Outcomes of the patients based on their body mass index. Data are represented as frequency and Kruskal-Wallis.

Outcomes (n, %)	BMI			P-value
	30-34.9	35-39.9	≥40	
Leaking (18, 3.21%)	7	5	6	0.016
Stenosis, twists or kinks (2, 0.36%)	0	1	1	0.987
Venous thromboembolism (3, 0.53%)	0	1	2	0.718
Gallstone disease (16, 2.85%)	6	7	3	0.003
Marginal ulceration (1, 0.18%)	0	0	1	0.518
Perforation (1, 0.18%)	0	1	0	0.503
Small bowel obstruction (2, 0.36%)	1	1	0	0.123
Internal hernia (10, 1.78%)	3	2	5	0.861
Readmission (12, 2.14%)	2	4	6	0.798

The outcomes of patients based on chronic illness revealed two significant differences only, where the internal hernia was significantly higher among patients with diabetes mellitus, hypertension, and dyslipidemia (P=0.001). Additionally, readmission was significantly reported among diabetic patients (P=0.002) (Table 5).

**Table 5:** Outcomes of the patients based on their chronic illnesses. Data are represented as frequency and Kruskal-Wallis.

Outcomes	Chronic illness												P-value
	DM	HTN	Dyslipidemia	Asthma	HF	A-fib	OSA	Stroke	CAD	Hemodialysis	GERD	CKD	
Leaking	7	6	4	0	1	0	3	0	1	0	2	0	0.775
Stenosis, twists, or kinks	0	0	0	0	0	0	2	0	0	0	0	0	0.174
Venous thromboembolism	2	1	0	0	0	0	0	0	0	1	0	0	0.405
Gallstone disease	3	6	8	1	1	1	5	0	1	0	0	1	0.894
Marginal ulceration	0	0	0	0	0	0	1	0	0	0	0	0	0.646
Perforation	0	0	0	0	0	0	1	0	0	0	0	0	0.646
Small bowel obstruction	1	0	1	0	0	0	0	0	0	0	0	0	0.997
Internal hernia	4	4	4	1	0	0	2	1	0	0	2	0	0.001
Readmission	5	2	3	1	1	0	3	1	1	0	2	0	0.002

A comprehensive investigation was conducted to examine the influence of various factors on the outcomes of different obesity categories. Concerning diabetes mellitus, it was observed that a higher proportion of patients with class I, II, and III obesity reported leaking, gallstone disease, and internal hernia, respectively. Additionally, readmission rates were equally reported among individuals with class II and III obesity. Patients with hypertension and class I obesity exhibited a higher incidence of leaking, while those with class II obesity had a higher prevalence of VTEs, gallstone disease, and internal hernia. Similarly, readmission rates were equally reported among patients with class II and III obesity in this subgroup.

Moreover, patients with dyslipidemia and class I or II obesity experienced a higher occurrence of gallstone disease, whereas those with class III obesity reported a higher prevalence of internal hernia and readmission. As for the impact of asthma, three patients suffered from complications. One (4.8%) patient with class I obesity experienced gallstone disease, one (4.8%) patient with class III reported an internal hernia, and another one (4.8%) patient required readmission. Three patients with a history of heart failure developed complications. One patient (7.1%) with class I obesity experienced gallstone disease, one (7.1%) patient with class III obesity reported a leaking, and another one (7.1%) patient required readmission. Only one (25%) patient with A-fib-developed complications and belonged to class I obesity and reported gallstone disease. Among patients with OSA, gallstone disease was more commonly observed in individuals with class I and II obesity. Notably, there were two patients with stroke and class III obesity, with one (20%) experiencing an internal hernia and another one (20%) requiring readmission. In terms of CAD, one (33.3%) patient with class II obesity complained of gallstone disease, and one (33.3%) patient with class III obesity required readmission. Among patients on hemodialysis, one (100%) patient in the class II obesity category developed VTE. In patients with GERD, two (13.3%) individuals experienced internal hernia, two (13.3%) patients with leaking belonged to the class I obesity category, and two (13.3%) patients required readmission, with one belonging to class I obesity and another to class III obesity. Finally, only one (12.5%) patient with CKD and class I obesity had gallstone disease.

## Discussion

The utilization of bariatric surgery has witnessed an upward trend, with the most commonly performed procedure being LSG, accounting for 46% of all interventions [10]. The adverse outcomes of LSG encompass mortality and complications, which can be further classified into two main categories. Early complications occurring within 30 days of the procedure encompass bleeding, intraabdominal abscess, wound infection, and acute pancreatitis. Late complications, on the other hand, manifest after 30 days post-surgery and include gastric stenosis and GERD [3]. This study aimed to investigate the outcomes of LSG, including complications, mortality rate, and readmission.

In this study, there was a higher prevalence of female subjects, constituting 67.4% of the cohort. The patients exhibited various comorbidities, such as OSA, hypertension, diabetes mellitus, and dyslipidemia. A similar study conducted in Egypt on 140 obese patients who underwent LSG also reported a higher proportion of females (66%) and observed comorbidities of diabetes mellitus type 2, hypertension, and hyperlipidemia [11]. In the present study, a wide range of outcomes was observed, including gallstone disease, internal hernia, and leaks as significant complications.

A prospective study involving 156 LSG patients revealed a predominance of female patients (74.3%) and reported no mortality [12], which aligns with our findings of a higher proportion of female patients and zero mortality during the study period. The mortality rate associated with LSG varies and ranges between 0.18% and 0.27%, depending on age, sex, and comorbidities [13]. Furthermore, a previous study involving 218 obese LSG patients reported no mortality or readmission [14], whereas in our study, 2.1% of patients required readmission. Another study from Saudi Arabia, which included 301 gastric sleeve surgery patients, reported a readmission rate of 8%, with higher rates observed in older patients and those with class I obesity, diabetes, and OSA [15]. It is worth noting that readmission rates in our cohort were higher among class III obesity patients, although the difference was not statistically significant.

Conversely, readmission was significantly higher among patients with diabetes mellitus compared to those with other comorbidities. Additionally, among class III obesity patients, readmission rates were higher for those with dyslipidemia, asthma, heart failure, OSA, stroke, CAD, and GERD. However, due to the limited number of cases presenting comorbidities and complications, it was challenging to assess the precise impact of these comorbidities on readmission.

Various risk factors have been identified in predicting overall complications after LSG, including male gender, older age, higher BMI, smoking, and comorbidities, such as hypertension, diabetes, OSA, liver disease, and depression [16]. In our study, patients with hypertension, dyslipidemia, and diabetes were significantly associated with internal hernia, and diabetes was significantly associated with readmission. Moreover, based on obesity classes, diabetes mellitus was linked to leaking, gallstone disease, and internal hernia in class I, II, and III obesity, respectively. Hypertensive patients with class I obesity demonstrated a higher incidence of leaking, while hypertensive patients with class II obesity experienced VTE, gallstone disease, and internal hernia. Liver disease and depression were not reported in our study, and the assessment of predictors for LSG outcomes was challenging due to the presence of various comorbidities with limited case numbers.

A study involving 610 LSG patients reported early and late complications in 5.74% and 1.64% of patients, respectively. Independent predictors of late complications occurring at or after 30 days post-surgery were smoking, coexistence of hiatal hernia, and peptic ulcer disease. Smoking and hypercholesterolemia were identified as independent risk factors for early complications [17].

Another study from India, comprising 424 LSG patients, reported early complications such as bleeding, staple line leak, deep venous thrombosis, and 30-day mortality. Late complications included new-onset GERD [18]. In other studies, stenosis occurs among 0.69-2% of patients who underwent sleeve gastrectomy, whereas the rate of bleeding is higher in incidence as it may occur among 11% of the patients [19]. In our study, there were no cases of bleeding, GERD, or mortality, whereas only 0.5% reported VTE. The rate of GERD among patients was based on the medical history of patients, but none reported GERD post-surgery. Additionally, stenosis was of low incidence and was found among only 0.4% of our patients.

The limitations of this study included that we did not investigate the predictors of LSG complications and the significant impact of comorbidities on LSG outcomes. However, this was due to the various comorbidities and outcomes reported, as well as the few studies that experienced such complications. A larger sample size and further studies would aid in determining outcome predictors.

## Conclusions

Laparoscopic sleeve gastrectomy stands as a safe surgical modality for addressing the issue of obesity. This study has exhibited minimal incidence of adverse events, notably an absence of bleeding or aspiration pneumonia and a complete absence of reported mortalities. Moreover, the frequency of complications displayed a positive correlation with the severity of obesity, and individuals presenting with pre-existing comorbidities also demonstrated a notably low likelihood of experiencing such complications. To advance our understanding in this domain, we advocate for future investigations encompassing more expansive cohorts, as well as the identification of outcome predictors.

## References

[1] Rahimuddin NO, Alanazi WK, Alabdulhadi RM (2022). Early and late complication of sleeve gastrectomy. Int J Community Med Public Health.

[2] Alamoudi MA, Softah AA, Softah AA (2018). Outcome of bariatric surgery in Saudi Arabia: a systematic review. Int J Adv Res.

[3] Woźniewska P, Diemieszczyk I, Hady HR (2021). Complications associated with laparoscopic sleeve gastrectomy - a review. Gastroenterology Rev.

[4] Mahfouz ME, Altowairqi A, Altowairqi H (2016). Impact of sleeve gastrectomy on weight loss, comorbidities of obesity, and quality of life in Saudi Arabia. Egypt J Hosp Med.

[5] Welbourn R, Hollyman M, Kinsman R (2019). Bariatric surgery worldwide: baseline demographic description and one-year outcomes from the fourth IFSO global registry report 2018. Obes Surg.

[6] Or Koca A, Buluş H (2023). Metabolic outcomes in the first year after laparoscopic sleeve gastrectomy: a high-volume single-center experience. Eur Rev Med Pharmacol Sci.

[7] Wojciak PA, Pawłuszewicz P, Diemieszczyk I (2020). Laparoscopic sleeve gastrectomy: a study of efficiency in treatment of metabolic syndrome components, comorbidities and influence on certain biochemical markers. Videosurgery Miniinv.

[8] Karmali S, Schauer P, Birch D, Sharma AM, Sherman V (2010). Laparoscopic sleeve gastrectomy: an innovative new tool in the battle against the obesity epidemic in Canada. Can J Surg.

[9] Moulla Y, Lyros O, Blüher M, Simon P, Dietrich A (2018). Feasibility and safety of bariatric surgery in high-risk patients: a single-center experience. J Obes.

[10] Alizadeh RF, Li S, Gambhir S (2019). Laparoscopic sleeve gastrectomy or laparoscopic gastric bypass for patients with metabolic syndrome: an MBSAQIP analysis. Am Surg.

[11] Elsherif A (2016). Outcomes after laparoscopic sleeve gastrectomy for morbidly obese patients. J Med Res Inst.

[12] Hoyuela C (2017). Five-year outcomes of laparoscopic sleeve gastrectomy as a primary procedure for morbid obesity: a prospective study. World J Gastrointest Surg.

[13] Chivot C, Robert B, Lafaye N (2013). Laparoscopic sleeve gastrectomy: imaging of normal anatomic features and postoperative gastrointestinal complications. Diagn Interv Imaging.

[14] Hans PK, Guan W, Lin S, Liang H (2018). Long-term outcome of laparoscopic sleeve gastrectomy from a single center in mainland China. Asian J Surg.

[15] Ahmed A, AlBuraikan D, ALuqbil B, AlJohi W, Alanazi W, AlRasheed B (2017). Readmissions and emergency department visits after bariatric surgery at Saudi Arabian hospital: the rates, reasons, and risk factors. Obes Facts.

[16] Husain F, Jeong IH, Spight D, Wolfe B, Mattar SG (2018). Risk factors for early postoperative complications after bariatric surgery. Ann Surg Treat Res.

[17] Głuszyńska P, Diemieszczyk I, Szczerbiński Ł, Krętowski A, Major P, Razak Hady H (2022). Risk factors for early and late complications after laparoscopic sleeve gastrectomy in one-year observation. J Clin Med.

[18] Garg H, Aggarwal S, Misra MC, Priyadarshini P, Swami A, Kashyap L, Jaiswal R (2017). Mid to long term outcomes of laparoscopic sleeve gastrectomy in Indian population: 3-7 year results - a retrospective cohort study. Int J Surg.

[19] Lim R, Beekley A, Johnson DC, Davis KA (2018). Early and late complications of bariatric operation. Trauma Surg Acute Care Open.

